# Clinical utility of ultrasonography in pediatric and adolescent gynecology: retrospective review of 1313 ultrasound examinations

**DOI:** 10.1002/uog.29155

**Published:** 2025-02-02

**Authors:** N. Cooper, H. Meehan, K. Linton‐Reid, J. Barcroft, J. Danin, M. Seah, S. Sadigh, N. Bharwani, S. Sur, C. Fotopoulou, C. Landolfo, J. Yazbek, S. Saso, D. Timmerman, T. Bourne, M. Al‐Memar

**Affiliations:** ^1^ Queen Charlotte's and Chelsea Hospital, West London Gynaecological Cancer Centre Imperial College Healthcare NHS Trust London UK; ^2^ Department of Metabolism, Digestion and Reproduction Imperial College London London UK; ^3^ KU Leuven Leuven Belgium; ^4^ Division of Surgery and Cancer Faculty of Medicine, Imperial College London London UK; ^5^ Imaging, Imperial College Healthcare NHS Trust London UK

**Keywords:** adolescent, diagnostic imaging, pediatric, reproductive medicine, sonography

## Abstract

**Objectives:**

Ultrasound is the first‐line imaging modality of the pelvis in the pediatric and adolescent gynecology (PAG) population. Ultrasound findings in pre‐ and postpubertal PAG patients differ from those in adults. Diagnostic models for adnexal pathology have not been validated in this cohort. The primary aim of this study was to evaluate normative findings and the incidence of pathology in this cohort. The secondary aim was to assess the performance of expert opinion alone, as well as using retrospective application of the International Ovarian Tumor Analysis (IOTA) simple rules (SRs) and benign descriptors (BDs) in those found to have an adnexal mass.

**Methods:**

This was a retrospective review of pelvic ultrasound examinations performed in patients < 18 years of age from January 2017 to July 2021 in one expert center in the UK. Analysis was performed on three age groups: neonatal (aged < 1 year), premenarchal (aged ≥ 1 year) and postmenarchal. The study was locally approved as an audit (GRM_082). Expert review of images of ovarian masses was performed using retrospective application of the IOTA‐SRs and IOTA‐BDs.

**Results:**

In total, data on 1429 pelvic ultrasound examinations were retrieved, of which 116 were excluded, resulting in the inclusion of 1313 ultrasound images (1145 patients). The median age at the first ultrasound scan was 2 days after birth in the neonatal group (*n* = 20), 8.8 years in the premenarchal group (*n* = 124) and 16.1 years in the postmenarchal group (*n* = 961). The status of menarche was unknown in a further 40 patients. Normative ultrasound findings were in keeping with those in the existing literature. Uterine anomalies were seen in 14 (1.2%) patients. Endometrial pathology was rare, with five cases of gestational trophoblastic disease. The most frequent indication for ultrasound scan for each group were a known medical condition in neonates (*n* = 11 (55.0%)), suspected precocious puberty in premenarchal girls (*n* = 38 (30.6%)) and abnormal vaginal bleeding in postmenarchal girls (*n* = 504 (52.4%)). Polycystic ovarian appearances were described in 150 (15.6%) postmenarchal girls. Adnexal pathology was identified in 102 (8.9%) participants on initial ultrasound: four neonates, three premenarchal and 95 postmenarchal patients. Benign cystadenomas and hemorrhagic cysts were the most common adnexal mass type in all groups. Final outcomes were available for 79/95 masses in the postmenarchal group, none of which were malignant. The IOTA‐SRs, IOTA‐BDs, expert opinion and standard ultrasound reporting could characterize as benign 96.2%, 87.3%, 98.7% and 77.2% of the masses, respectively, all with a specificity of 100%. Eleven patients underwent 12 surgeries overall (three oophorectomies, six cystectomies and three cyst aspirations), with 11 out of 12 masses classified as benign based on retrospective expert assessment.

**Conclusions:**

Ultrasound is effective for assessment of the female pelvis in the PAG population. Adnexal masses are common, but few require surgical intervention and most resolve expectantly. The IOTA‐BDs and IOTA‐SRs maintain their performance in this population. Larger studies are required for the prospective validation of diagnostic models which may aid a fertility‐sparing approach to care. © 2024 The Author(s). *Ultrasound in Obstetrics & Gynecology* published by John Wiley & Sons Ltd on behalf of International Society of Ultrasound in Obstetrics and Gynecology.

## INTRODUCTION

Transabdominal ultrasonography is the most common ultrasound modality in the context of pediatric and adolescent gynecology (PAG), as it is easy to perform, does not use ionizing radiation and does not require sedation. Transvaginal and transrectal ultrasound can also be performed in cases in which transabdominal ultrasound is inadequate^1^, ^2^, ^3^. The sonographic appearance of the internal genitalia changes throughout development in response to fluctuations in endogenous activity of the hypothalamic–pituitary–gonadal axis and in response to exogenous maternal estrogen *in utero*. Therefore, pathology should be interpreted in the context of age‐related physiology^4^, ^5^.

The prevalence of ovarian malignancy is reportedly 3.7–25.2% in the pediatric and adolescent population; however, this is probably an overestimate^6^. Most malignant tumors are either malignant ovarian germ cell tumors or juvenile granulosa cell tumors, in contrast to the predominance of epithelial ovarian cancers in adults. The proportion of malignancy is greatest in masses identified premenarche^6^, ^7^, ^8^, ^9^.

Existing ultrasound indices have been validated on small populations of children and adolescents using combinations of clinical and ultrasonographic features, such as size of mass, presence of septae and vascularity^6^, ^10^, ^11^, ^12^, ^13^, ^14^, ^15^. No model has been externally validated prospectively in large studies and maintained its performance to warrant recommendation for use in day‐to‐day care^16^. Furthermore, there is over‐representation of malignancy and masses managed surgically in the cohorts tested previously; thus, these indices may not represent the reality of clinical practice^10^, ^11^, ^12^, ^13^, ^14^, ^15^.

The International Ovarian Tumor Analysis (IOTA) benign simple descriptors (BDs), simple rules (SRs) and the ADNEX (Assessment of Different NEoplasias in the adneXa) models have been widely validated externally; however, none has been validated for use below 18 years of age^16^, ^17^, ^18^, ^19^, ^20^, ^21^, ^22^. Additionally, while the ADNEX model allows input from 14 years of age, it is not recommended for use in PAG patients. Confidence in the characterization of adnexal masses using ultrasound alone may avoid the need for additional imaging modalities that require intervention in this age group, such as sedation or anesthesia to accommodate magnetic resonance imaging (MRI) or computed tomography.

We aimed to describe the normative findings of pelvic ultrasound in the PAG population and establish the incidence of pathology. Our secondary aim was to assess the performance of IOTA‐SRs and IOTA‐BDs prediction models, as well as expert opinion, in cases with an adnexal mass.

## METHODS

This was a retrospective review of pelvic transabdominal and transvaginal ultrasound scans performed in children and adolescents aged < 18 years, from 1 January 2017 to 31 July 2021 at Imperial College Healthcare NHS Trust, London, UK, an IOTA‐certified ultrasound center and European Society of Gynaecological Oncology (ESGO) center of excellence for gynecological cancer. This timeframe was chosen to ensure that at least 12 months had elapsed since a patient's previous scan to ensure that outcomes could be recorded for conservatively managed patients. The study was registered and approved as an audit at Imperial College Healthcare NHS Trust (registration number: GRM_082).

Imaging studies were identified via an electronic search of PACS Imaging Software (Philips Medical Systems, Solingen, Germany) database. Clinical data were obtained from electronic patient records (Cerner; Kansas City, MO, USA). Ultrasound scan reports were screened to extract predefined data criteria. When an ultrasound scan report flagged an adnexal mass, the ultrasound images of the adnexal masses were reviewed by two IOTA‐certified gynecologists (M.A.‐M. (European Federation For Ultrasound in Medicine and Biology (EFSUMB) Level 3) and N.C. (EFSUMB Level 2)) and characterized using IOTA terminology^23^. The expert impression of the nature of the mass was recorded, and the modified BDs and SRs were applied retrospectively. The ADNEX model was not applied owing to the model not allowing entry below 14 years of age. Images for which a diagnosis was not made were reviewed by a third expert examiner (T.B. (EFSUMB Level 3)). For cases in which serial ultrasound was performed on the same patient, measurements of gynecological organs, clinical presentation data and scan findings were obtained from the first scan only.

Outcomes were predefined as per previous IOTA study methodology^24^. If surgery was performed, a mass was considered benign or malignant based on pathology. If surgery was not performed, a mass was considered benign if it resolved (i.e. it was not seen on a subsequent scan) based on pattern recognition (i.e. subjective assessment of the ultrasound examiner) or if no change in the mass was seen between two consecutive scans. There was no standardized follow‐up timeframe for masses, reflective of existing national practice. Diagnosis of a uterine anomaly or suspected endometrial or myometrial pathology was based on the assessment of the ultrasound examiner.

All female patients identified from the database search who were < 18 years of age and underwent an initial transabdominal or transvaginal ultrasound scan of the pelvis were included. The following cases were excluded: non‐molar pregnancy, imaging of non‐gynecological organs, duplicate imaging, incorrect coding (i.e. a scan incorrectly labeled as an ultrasound scan of the pelvis), failure to attend follow‐up and incomplete imaging (i.e. a pelvic ultrasound scan in which dedicated imaging of the female reproductive organs was not performed).

### Statistical analysis

Statistical analysis was carried out using SPSS version 27 (IBM Corp., Armonk, NY, USA) and R version 4.1.2 (www.r‐project.org) in three age groups: neonatal (aged < 1 year), premenarchal (aged ≥ 1 year) and postmenarchal. Data for patients with an unknown menarchal status are also presented. To evaluate the performance of the IOTA models, statistical methods were selected to account for both categorical and continuous data types effectively. Wilson's method was used to determine 95% CIs. For categorical variables, such as patient age classifications (neonatal, premenarchal and postmenarchal) and lesion characteristics, the chi‐square test or Fisher's exact test was used to explore associations with clinical outcomes, allowing for a nuanced analysis of distributional differences across age groups. Continuous variables, including lesion size and vascularity, were analyzed using independent *t*‐tests for pairwise comparisons and ANOVA was used to compare means across the three age groups, providing a comprehensive understanding of clinical measurement trends and variations.

## RESULTS

Data were retrieved for a total of 1429 pelvic ultrasound scans performed between January 2017 and July 2021 in girls aged < 18 years. Following exclusion of 116 scans, 1313 scans were included in the final analysis from a total of 1145 patients. The patients were stratified into three groups: neonates aged < 1 year (*n* = 20 patients, 21 scans); premenarchal girls aged ≥ 1 year (*n* = 124 patients, 134 scans); and postmenarchal girls (*n* = 961 patients, 1114 scans). Menarchal status was unknown in 40 cases (44 scans).

### Cohort characteristics

The median age at the time of first ultrasound was 2 (interquartile range (IQR), 0–105) days after birth for the neonatal group, 8.8 (IQR, 3.9–14.5) years for the premenarchal group and 16.1 (IQR, 14.8–17.2) years for the postmenarchal group. The number of scans per patient, urgency of referral, ultrasound modality and primary operator are summarized in Table 1. Most patients required only one scan. Normative imaging findings are reported in Table S1. All patients underwent first‐line ultrasound, except one patient who had an X‐ray first.

**Table 1:** Characteristics of ultrasound examinations in neonatal (aged < 1 year), premenarchal (aged ≥ 1 year) and postmenarchal groups, as well as those in whom menarche was not documented

Characteristic	Neonatal (*n* = 20)	Premenarchal (*n* = 124)	Postmenarchal (*n* = 961)	Menarche not documented (*n* = 40)
Priority				
Inpatient				
Urgent	4 (20.0)	6 (4.8)	43/956 (4.5)	0 (0)
Routine	1 (5.0)	1 (0.8)	6/956 (0.6)	0 (0)
Outpatient				
2‐week wait	1 (5.0)	2 (1.6)	4/956 (0.4)	0 (0)
Urgent	3 (15.0)	16 (12.9)	65/956 (6.8)	0 (0)
Routine	11 (55.0)	99 (79.8)	838/956 (87.7)	40 (100)
Initial imaging modality				
TAS	20 (100)	124 (100)	894 (93.0)	37 (92.5)
TAS + TVS	0 (0)	0 (0)	60 (6.2)	3 (7.5)
TVS	0 (0)	0 (0)	6 (0.6)	0 (0)
X‐ray	0 (0)	0 (0)	1 (0.1)	0 (0)
Primary operator				
Radiologist				
Consultant	9 (45.0)	85 (68.5)	389 (40.5)	31 (77.5)
Specialty registrar	5 (25.0)	11 (8.9)	106 (11.0)	1 (2.5)
Level unspecified	4 (20.0)	6 (4.8)	54 (5.6)	4 (10.0)
Sonographer	2 (10.0)	21 (16.9)	410 (42.7)	4 (10.0)
Other	0 (0)	1 (0.8)	2 (0.2)	0 (0)
Number of scans performed per patient	1 (1–2)	1 (1–3)	1 (1–6)	1 (1–2)
1	19 (95.0)	113 (91.1)	840 (87.4)	38 (95.0)
2	1 (5.0)	10 (8.1)	103 (10.7)	2 (5.0)
3	0 (0)	1 (0.8)	10 (1.0)	0 (0)
4	0 (0)	0 (0)	4 (0.4)	0 (0)
5	0 (0)	0 (0)	2 (0.2)	0 (0)
6	0 (0)	0 (0)	2 (0.2)	0 (0)

Data are given as *n* (%), *n*/*N* (%) or median (range).

TAS, transabdominal ultrasound; TVS, transvaginal ultrasound.

The indications for initial referral are summarized in Table 2. The most common cause for referral in neonates was a known medical condition (*n* = 11 (55.0%)), in premenarchal girls it was suspected precocious puberty (thelarche or adrenarche) (*n* = 38 (30.6%)) and in postmenarchal girls it was abnormal vaginal bleeding (PVB) (*n* = 504 (52.4%)), including oligomenorrhoea, menorrhagia and other bleeding abnormalities, such as intermenstrual bleeding.

**Table 2:** Indication for referral for ultrasound in neonatal (aged < 1 year), premenarchal (aged ≥ 1 year) and postmenarchal groups, as well as those in whom menarche was not documented

Indication for referral	Neonatal (*n* = 20)	Premenarchal (*n* = 124)	Postmenarchal (*n* = 961)	Menarche not documented (*n* = 40)
Abnormal vaginal bleeding	1 (5.0)	11 (8.9)	504 (52.4)	—
Menorrhagia	0 (0)	0 (0)	206 (21.4)	—
Oligomenorrhea	0 (0)	0 (0)	263 (27.4)	—
Other*	1 (5.0)	11 (8.9)	35 (3.6)	—
Abdominal pain	0 (0)	10 (8.1)	187 (19.5)	6 (15.0)
Primary amenorrhea	0 (0)	31 (25.0)	0 (0)	0 (0)
Secondary amenorrhea	0 (0)	0 (0)	63 (6.6)	0 (0)
Known medical condition	11 (55.0)	4 (3.2)	29 (3.0)	0 (0)
Change in bowel habit	0 (0)	0 (0)	1 (0.1)	0 (0)
Suspected precocious puberty†	0 (0)	38 (30.6)	0 (0)	0 (0)
Uncertain menarchal status	0 (0)	2 (1.6)	3 (0.3)	0 (0)
Hirsutism	0 (0)	1 (0.8)	20 (2.1)	1 (2.5)
Other	8 (40.0)	23 (18.5)	85 (8.8)	2 (5.0)
Not documented	0 (0)	4 (3.2)	69 (7.2)	31 (77.5)

Data are given as *n* (%).

* Intermenstrual, postcoital or prepubertal bleeding.

† Thelarche or adrenarche.

### Uterus

Uterine characteristics are summarized in Table 3. Congenital uterine anomalies were diagnosed by the scanning clinician in 14 (1.2%) patients and comprised congenital absence of the uterus (*n* = 5), subseptate uterus (*n* = 4), arcuate uterus (*n* = 2), septate uterus (*n* = 1), bicornuate uterus (*n* = 1) and uterus didelphys (*n* = 1). Menarchal status was unknown for one of these patients. Reported endometrial findings in 29 patients were: uterine polyps (*n* = 10), suspected endometrial hyperplasia (based on an endometrial thickness of > 10 mm) (*n* = 10), gestational trophoblastic disease (*n* = 5), cystic intracavity lesions (*n* = 2), free fluid in the endometrial cavity (*n* = 1) and atrophic endometrium for age (*n* = 1). None of these patients required further investigation or hysteroscopic assessment of the endometrial cavity after consultation with a gynecologist, because clinical correlation did not warrant invasive investigation or subsequent ultrasound demonstrated resolution of the findings. Of the five patients diagnosed with gestational trophoblastic disease, three had a grossly enlarged uterus.

**Table 3:** Uterine findings and ultrasound approach for visualization of uterus in neonatal (aged < 1 year), premenarchal (aged ≥ 1 year) and postmenarchal groups

Characteristic	Neonatal (*n* = 20)	Premenarchal (*n* = 124)	Postmenarchal (*n* = 961)
Uterine appearance			
Normal	18 (90.0)	114 (91.1)	935 (97.3)
Abnormal	1 (5.0)	1 (0.8)	14 (1.5)
No uterus	0 (0)	3 (2.4)	2 (0.2)
Inadequate visualization	1 (5.0)	6 (4.8)	10 (1.0)
Endometrial pathology			
None	19/19 (100)	112/114 (98.2)	922/949 (97.2)
Present	0/19 (0)	2/114 (1.8)	27/949 (2.8)
Congenital uterine anomaly			
None	20 (100)	121 (97.6)	951 (99.0)
Present	0 (0)	3 (2.4)	10 (1.0)
Visualization of uterus per approach			
Adequate visualization			
TAS	19 (95.0)	115/121 (95.0)	882/958 (92.1)
TVS	0 (0)	0/121 (0)	6/958 (0.6)
TAS + TVS	0 (0)	0/121 (0)	60/958 (6.3)
Inadequate visualization			
TAS	1 (5.0)	6/121 (5.0)	10/958 (1.0)
TVS	0 (0)	0/121 (0)	0/958 (0)
TAS + TVS	0 (0)	0/121 (0)	0/958 (0)

Data are given as *n* (%) or *n*/*N* (%).

TAS, transabdominal ultrasound; TVS, transvaginal ultrasound.

### Ovaries and adnexa

Characteristics of the ovaries and ovarian cysts are summarized in Tables 4 and S2, respectively. A transabdominal ultrasound approach was adopted exclusively in neonatal and premenarchal patients. In neonates, both ovaries were visualized in 50% of cases, one ovary in 15% and neither was identified in 35%. Adnexal masses were visualized in four (20%) cases. Upon expert review, one mass was determined to be a hemorrhagic cyst and two were presumed to be benign cystadenomas. One mass was not classifiable on ultrasound; however, it could be described as unilocular with anechoic or low‐level content and a thick posterior cyst wall that was suspected to be a benign adnexal lesion initially but was found subsequently to be a hydrocolpos on MRI.

**Table 4:** Ovarian findings and ultrasound approach for visualization of ovaries in neonatal (aged < 1 year), premenarchal (aged ≥ 1 year) and postmenarchal groups

Characteristic	Neonatal (*n* = 20)	Premenarchal (*n* = 124)	Postmenarchal (*n* = 961)
Ovarian volume (mL)			
Right ovary	2.5 (0.6–5.9)	4.2 (0.3–15.5)	10.1 (0.5–132.0)
Left ovary	3.0 (0.1–5.0)	4.8 (0.1–25.4)	9.2 (0.6–122.0)
Ovaries visualized			
Both	10 (50.0)	90 (72.6)	830 (86.4)
Left only	1 (5.0)	1 (0.8)	32 (3.3)
Right only	2 (10.0)	14 (11.3)	65 (6.8)
Neither	7 (35.0)	19 (15.3)	34 (3.5)
Cyst visualized			
Yes	4 (20.0)	3 (2.4)	95 (9.9)
No	16 (80.0)	121 (97.6)	866 (90.1)
Polycystic morphology			
Yes	0 (0)	6 (4.8)	150 (15.6)
No	20 (100)	118 (95.2)	811 (84.4)
Visualization of both ovaries per approach			
Adequate visualization			
TAS	10 (50.0)	90 (72.6)	765 (79.6)
TVS	0 (0)	0 (0)	6 (0.6)
TAS + TVS	0 (0)	0 (0)	59 (6.1)
Inadequate visualization			
TAS	10 (50.0)	34 (27.4)	130 (13.5)
TVS	0 (0)	0 (0)	0 (0)
TAS + TVS	0 (0)	0 (0)	1 (0.1)

Data are given as mean (range), *n* (%) or *n*/*N* (%).

TAS, transabdominal ultrasound; TVS, transvaginal ultrasound.

In the premenarchal group, both ovaries were visualized adequately in 72.6% of cases, only one ovary was identified in 12.1% of cases and neither was identified in 15.3% of cases. ‘Polycystic’ appearance of one or both of the ovaries was noted in the scan reports in six (4.8%) patients. Ovarian masses were identified in three (2.4%) patients. The first mass appeared avascular and solid on ultrasound and was found to be a calcified ischemic ovary at surgery. Upon retrospective review, this was identified as a congenital mass that was difficult to classify. On postnatal ultrasound immediately after birth, the mass was described as unilocular with a thick cyst wall and possible dense hemorrhagic content. It was suspected to be malignant, but subsequent tumor markers were normal; thus, the neonate was managed expectantly for 13 months, at which point a diagnostic laparoscopy was recommended in view of ‘persistent solid tissue of uncertain etiology’. The solid mass represented a congenital ovarian torsion with subsequent ischemia and calcification of the ovary. The second mass was described as ‘uncertain’ in nature and thought to be a possible right ovarian teratoma (unilocular with mixed content) upon review by a consultant radiologist, but the patient was referred externally to a national pediatric hospital as a possible recurrence of a sacral teratoma, thus, the final diagnosis is unknown. The third patient was found to have a unilocular mass with anechoic content, seen separately to the uterus. At first, this was thought to be a possible adnexal mass or hydrocolpos, however, it had resolved spontaneously at MRI and no further management was required. A further two (5.0%) ovarian cysts were identified in the cohort of 40 patients whose menarchal status was unknown.

In postmenarchal patients, both ovaries were identified in 79.6% of cases when only a transabdominal approach was used. In cases in which both a transvaginal and transabdominal approach was used, both ovaries were identified in a further 59 cases. In six postmenarchal cases in which a transvaginal approach was implemented exclusively to visualize the uterus and ovaries, both ovaries were identified in all cases. Polycystic appearance of one or both ovaries – based on the subjective impression of the sonologist – was described in the ultrasound scan report in 150 (15.6%) patients. An ovarian cyst was identified on the first scan in 95 (9.9%) patients. The median age at cyst diagnosis was 15.7 (IQR, 11.1–17.9) years. Most cysts were unilocular (*n* = 82 (86.3%)), with a median diameter of 43.5 (IQR, 13.1–108.0) mm. Based on expert impression, the most common types of cysts were presumed hemorrhagic cyst (*n* = 35 (36.8%)), benign cystadenoma (*n* = 34 (35.8%)) or dermoid cyst (*n* = 6 (6.3%)). Eleven of these 95 patients (11.6%) were discharged after their first scan and 19 (20.0%) were referred to gynecology. Almost two‐thirds of postmenarchal patients with an ovarian cyst underwent a repeat ultrasound scan (*n* = 62 (65.3%)), in which most (*n* = 40 (64.5%)) cysts were noted to have resolved spontaneously, 11 (17.7%) remained unchanged and only three (4.8%) appeared to have increased in size.

After retrieval of data, analysis of the performance of the IOTA‐SRs, IOTA‐BDs and expert opinion alone was conducted only in postmenarchal patients owing to the small number of masses seen in premenarchal patients. In the postmenarchal population, final outcomes were known for 83.2% (79/95) of masses, none of which were malignant; these are described in Table S3. The IOTA‐SRs were applicable in 96.2% (76/79) of masses and were unable to classify 3.8% (3/79) of masses, achieving a specificity of 100% (95% CI, 95.5–100%) and a negative predictive value (NPV) of 1.0 (95% CI, 0.955–1.0). The IOTA‐BDs were applicable in 87.3% (69/79) of masses and were not applicable in 12.7% (10/79), demonstrating a specificity of 100% (95% CI, 94.6–100%) and NPV of 1.0 (95% CI, 0.946–1.0). Expert opinion alone could characterize 98.7% (78/79) of masses; one (1/79 (1.3%)) mass was not classified. Expert opinion performed with a specificity of 100% (95% CI, 96.1–100%) and a NPV of 1.0 (95% CI, 0.961–1.0). Standard ultrasound reporting classified 77.2% of masses as benign and 22.8% (18/79) were not characterized, with a specificity of 100% (95% CI, 93.9–100%) and a NPV of 1.0 (95% CI, 0.939–1.0).

### Clinical correlation

Tables S4 and S5 provide details on the incidence of pathology for each indication for ultrasound examination.

#### Neonatal

Most neonatal referrals were made for a known medical condition (*n* = 11 (55%)) (Table 2). No congenital uterine anomalies were identified. The mean endometrial thickness in the neonatal group was < 2 mm, regardless of the indication for ultrasound, and no endometrial pathology was identified. Four ovarian cysts were identified and three of these were diagnosed antenatally. None of these cysts underwent immediate drainage or surgical management. One neonate had a subsequent MRI that confirmed that the adnexal mass was a hydrocolpos and the patient underwent incision of an imperforate hymen. Two neonates were followed up at a pediatric surgery outpatient clinic and were subsequently discharged. The remaining neonate was referred for repeat scans and was lost to follow‐up.

#### Premenarchal

The most frequent indication for referral for ultrasound in the premenarchal group was suspected precocious puberty (*n* = 38 (30.6%)), followed by primary amenorrhea (*n* = 31 (25.0%)) (Table 2). Premenarchal girls were significantly more likely to receive follow‐up compared with postmenarchal patients (*P* < 0.001). No ovarian cysts were identified in those referred for precocious puberty. When comparing age and classification of masses, ovarian cysts in younger patients were significantly more likely to not be classified definitively by the scanning clinician compared with older patients (t(98) = 3.31, *P* < 0.05).

In those referred for primary amenorrhea (*n* = 31), there were three cases of a uterine anomaly, representing 9.7% of these referrals. Two referrals for ‘uncertain menarchal status’ were for young girls with a single episode of vaginal menstrual‐like bleeding, for which the clinician or family were unsure if it represented menarche.

#### Postmenarchal

The most common indication for referral in the postmenarchal group was abnormal PVB (menorrhagia, oligomenorrhea or other vaginal bleeding) (*n* = 504 (52.4%)) (Table 2). Suspected endometrial pathology was found in 15 (3.0%) patients referred for abnormal uterine bleeding; however, only five of these patients were found to have proven pathology (gestational trophoblastic disease). The remaining 12 patients with suspected endometrial pathology were referred for indications other than abnormal PVB. A further 10 uterine anomalies were identified in postmenarchal patients who presented with abnormal PVB (*n* = 4), abdominal pain (*n* = 3) or other indications (*n* = 3). Mean endometrial thickness in patients with menorrhagia was similar to that in the overall cohort (7.0 mm *vs* 6.3 mm) and was within normal limits. This was similar for those with oligomenorrhea (6.5 mm *vs* 6.3 mm) and those with other causes of abnormal uterine bleeding (6.7 mm *vs* 6.3 mm). Unfortunately, the timing of the scan within the menstrual cycle was not always provided and so reported endometrial thicknesses are not cycle‐stage specific.

Adnexal pathology was identified in 95 postmenarchal girls on their first ultrasound scan. In these patients, 26 (27.4%) presented with menorrhagia and 23 (24.2%) presented with abdominal pain. Multifollicular appearance of the ovary was reported most commonly in patients referred with oligomenorrhea (68/150 (45.3%)), and this was described as ‘polycystic appearance’ by the reporting sonographer.

### Surgery

Indications for and type of surgical management for adnexal masses are summarized in Table 5. Twelve surgical procedures were performed in 11 patients. There was no significant difference in the rate of surgery between the age groups (*P* = 0.19) and no significant association between age of the patient and the decision for whether surgery was indicated (t(9.1) = −0.87, *P* = 0.40 (g = −0.28 (95% CI, −0.92 to 0.37))). Adnexal masses managed surgically were not significantly larger than those that were managed non‐surgically (t(5.03) = 1.64, *P* = 0.16 (g = 0.78 (95% CI, −0.29 to 1.80))). There were five cases of ovarian–adnexal torsion confirmed surgically, one of which was congenital. Oophorectomy was performed in 3/12 (25.0%) cases for ischemic necrosis secondary to torsion (*n* = 2) or due to the size of the cyst (*n* = 1). All masses with histology results available were benign. Surgery was performed by pediatric surgeons, gynecologists or gyneoncologists.

**Table 5:** Diagnosis, surgery details and histology for 12 surgical procedures performed in 11 patients

Surgical procedure	Diagnosis as given on US report	Presumed diagnosis on US*	Surgical findings	Operating team	Histology
Laparoscopic right ovarian cystectomy	Not classified	Hemorrhagic cyst	Large hemorrhagic simple right ovarian cyst, normal ovary, no torsion	Pediatric surgeons	No histology report available
Laparoscopy, drainage of ovarian cyst and adhesiolysis	Hemorrhagic cyst, torted ovary	Hemorrhagic cyst	7‐cm functional cyst on right, adhesions between ovary and rectum, no torsion	Gynecologists	Cyst fluid: benign content
Laparoscopic converted to open ovarian cystectomy	Dermoid cyst	Dermoid cyst	Operation notes unavailable	Gyneoncologists	Benign cystic teratoma
Laparoscopic right paratubal cystectomy	Not classified	Benign cystadenoma	Right paratubal cyst and tubal torsion	Gynecologists	No histology report available
Laparoscopic ovarian cyst drainage	Complex avascular cyst	Hemorrhagic cyst	Left ovarian simple cyst and ruptured hemorrhagic cyst	Gynecologists	No histology report available
Laparoscopic right salpingo‐oophorectomy	Dermoid cyst	Dermoid cyst	Operation notes unavailable	Gynecologists	No histology report available
Laparoscopic right oophorectomy	Indeterminate	Indeterminate	Torted right ovary	Pediatric surgeons	Benign calcified ischemic ovarian tissue
Laparoscopic right ovarian cystectomy	Mucinous cystadenoma	Benign cystadenoma	Large right ovarian cyst	Gyneoncologists	Benign mature cystic teratoma
Left salpingo‐oophorectomy and drainage of right ovarian cyst	Large septated cyst	Hemorrhagic cyst	Left ovarian torsion with necrosis of fimbriae	Pediatric surgeons	Benign calcified ischemic ovarian tissue
Laparoscopic left ovarian cystectomy	Not classified	Benign cystadenoma	Torted 5‐cm cyst in left ovary	Pediatric surgeons	Benign serous cystadenoma
Laparoscopy and drainage of hemorrhagic cyst	Complex mass, possible torsion	Hemorrhagic cyst	4‐cm right hemorrhagic cyst, no evidence of torsion	Dual specialty	No histology report available
Laparoscopic removal of ovarian cyst	Not classified	Paraovarian cyst	4‐cm paratubal cyst on long stalk, torted 2 cm from fimbrial end of tube, another separate 2‐cm cyst noted	Gynecologists	Benign serous cystadenoma

* According to retrospective expert opinion. US, ultrasound.

## DISCUSSION

Ultrasound is an effective modality for assessing the female pelvis in the PAG population. In our study population, mean measurements of the pelvic organs were consistent with published data. Uterine anomalies were identified on ultrasound in 14 patients. In the premenarchal population, a congenital uterine anomaly was identified in 2.4% of patients, all of whom presented with primary amenorrhea. Abdominal pain and abnormal vaginal bleeding were the most prominent symptoms in postmenarchal patients with congenital uterine anomalies. In a previous prospective series on ultrasound examination in adults and children, eight uterine anomalies were identified in 2065 consecutive scans, resulting in a prevalence of 3.87 per 1000 women^25^. The prevalence in our cohort is relatively higher, which may be because anomalies are likely to be identified around menarche.

Endometrial pathology was uncommon. A grossly thickened endometrium was confirmed in patients with gestational trophoblastic disease, which was probably over‐represented as our unit is the national center for gestational trophoblastic disease. In the remaining patients, reported endometrial pathology included suspected polyps and cystic spaces. None of these patients required hysteroscopic assessment, suggesting that repeat ultrasound alongside clinical correlation is sufficient. This is supported by the paucity of case reports of endometrial pathology in childhood^26^, ^27^.

Polycystic ovaries were reported in 15.6% of postmenarchal patients. The UK National Institute for Clinical Excellence guidance states that ultrasound should not be used for the diagnosis of polycystic ovary syndrome (PCOS) in adolescence, as multifollicular ovaries are physiological^28^. Incorrectly diagnosing an adolescent with PCOS can cause unnecessary medicalization, subsequent anxiety and implications throughout their reproductive lifespan. Therefore, using the term ‘multifollicular’ may be more appropriate when describing ovarian morphology in adolescents.

Adnexal masses were visualized on initial ultrasound in 9.9% of postmenarchal patients. Almost one‐quarter of this group presented with abdominal pain and most cysts were either benign ‘simple’ cystadenomas or hemorrhagic cysts. This supports the idea that ovulatory pain being related to functional cysts is a common clinical feature. Torsion remains a differential diagnosis. The absence of a cyst does not exclude ovarian torsion as a differential diagnosis, as adnexal torsion in those under 16 years of age involves a single ovary without an associated mass or cyst in up to 46% of cases^3^, ^4^. Clinical correlation of ultrasound findings and a patient's symptoms is vital. Current guidance supports detorsion of an ovary without oophorectomy unless the necrotic ovary ‘falls apart upon manipulation’^3^. In our cohort, 12 surgeries were performed in 11 patients and oophorectomy was performed in three instances, twice for torsion with ischemia and once for a large dermoid. Similarly, a 2021 review of 1783 children with ovarian torsion demonstrated a 22.5% rate of oophorectomy. Detorsion was not considered, which led to the recommendation of multidisciplinary working between gynecologists and pediatric surgeons^29^.

Although most masses in the PAG population are benign, many undergo surgery for fear of an underlying malignancy. There was inconsistent description of cysts in the data obtained for this study, with hemorrhagic cysts reported as ‘septated’ or ‘complex’. Indeed, some patients with cysts were referred for additional imaging, such as MRI for hemorrhagic cysts. Furthermore, three diagnostic laparoscopies were performed for a hemorrhagic cyst. Use of the IOTA terms may help to reduce the burden of additional imaging and unnecessary surgery^16^.

In cases in which uncertainty regarding the classification of a cyst remains, expert assessment is pertinent. Expert assessment was able to correctly characterize 98.7% of the masses. This is supported by the ESGO–European Society for Paediatric Oncology (SIOPE) guidance for the management of non‐epithelial ovarian cancers in the PAG population, which recommends that patients should be treated in centers with both adult and pediatric oncology expertise and that all patients should undergo abdominal and pelvic ultrasound^30^. This highlights the role of expert sonographers in all age groups.

Many small retrospective studies have attempted to apply models to classify adnexal masses in children. Such studies have shown a prevalence of malignancy of 5.1–22.6%, with most being non‐epithelial ovarian cancers, which is similar to the estimated overall prevalence of 3.7–25.2%^6^, ^8^, ^11^, ^12^, ^13^, ^14^, ^15^. An example of a model to classify adnexal masses is the Pediatric Risk of Malignancy Index^14^, which initially demonstrated promise, but had less discriminative capacity than originally published (cut‐off, > 7; sensitivity, 70.1%; specificity, 85.3%) and therefore is not recommended for use as a standalone diagnostic tool^15^. Another model is the morphology index by DePriest *et al*.^11^, which was prospectively validated and modified by Stankovic *et al*.^12^ to form the Ueland Index (sensitivity, 98.1%; specificity, 80.8%). Stankovic *et al*.^31^ prospectively assessed the ovarian crescent sign and applied the Ueland Index to 98 patients under 20 years of age. The ovarian crescent sign had a similar sensitivity to that of the Ueland Index (90% *vs* 90%) but it was less specific (72% *vs* 94%). As part of validating a PAG decision‐tree system, Stankovic *et al*.^32^ applied the IOTA‐SRs in masses that had been managed surgically, which performed with a specificity of 98% and a sensitivity of 90%. SRs were not applicable in 22% of these cases, which is higher than that of our cohort, where SRs were not applicable in only 3.8%. This is probably due to the exclusively benign nature of our cohort. The IOTA‐SRs describe five benign (‘B’) and five malignant (‘M’) ultrasonographic features of ovarian masses, if a mass displays soley B or M features, it can be classified as benign or malignant. When both B and M features are present, a mass is not classifiable. A tool capable of performing a risk assessment of less typical masses, such as those with both B and M features, is needed.

Indeed, the IOTA terms are known to be applicable in the fetal period, which may even allow accurate classification of masses antentally^33^. Retrospective application of the IOTA‐SRs and IOTA‐BDs in our population demonstrates promise, with specificities of 100%. This is similar to results from adult cohorts, in which SRs performed with a specificity of 96% (95% CI, 94–97%) and, where applicable, BDs were able to correctly classify 99.3% of benign masses^22^. Given that the BDs were not applicable in 12.7% of masses in our PAG cohort, a second‐line tool is needed. A potential solution for this would be the ADNEX model or two‐step strategy. Our data support prospective validation of IOTA models in the adolescent population.

### Strengths and limitations

This is an informative representation of a diverse PAG cohort representing multiple ethnic groups in a socioeconomically diverse London area. We present a mix of benign pathology, which probably represents the general population. Therefore, the results are probably applicable for physicians who work in non‐specialist services, and the outcomes are potentially generalizable.

The limitations of this study were the small number of masses with no cases of malignancy, meaning that our findings are limited to benign masses in postmenarchal patients. A large multicenter approach is required to capture cases of malignancy. Furthermore, the examiners relied on retrospective images that varied in quality, thus potentially skewing the specificity. At our unit there are pediatric radiologists and sonographers, but ultrasonographic assessment by a gynecologist is not routine. Finally, the decision for the division of patients into our chosen groups was pragmatic, as often there was limited referral information in scan requests and, thus, pubertal stage could not be extracted. Therefore, this does not reflect the reality of gradual changes seen through development.

### Conclusions

Ultrasound is a useful, safe and informative imaging modality for assessing the female gynecological organs in the PAG population. It can be a useful first‐line approach for assessment of the uterus and the diagnosis of anomalies, and it can also be used for assessing the endometrium and adnexa in the context of menstrual disturbance or abdominal pain. The reporting of adnexal masses is variable in this population, and uncertainty regarding the significance of a mass leads to unnecessary additional imaging and surgery. Standardized terminology should be used, and the IOTA‐BDs and IOTA‐SRs demonstrate some promise in the categorization of benign masses in this cohort. Until models are validated, expert assessment is warranted. We present one of the largest retrospective studies characterizing ultrasound findings in the PAG population, but prospective studies are required for the validation of diagnostic models.

## Supporting information


**Table S1** Findings of ultrasound examination of uterus and ovaries in neonatal (< 1 year), premenarchal (≥ 1 year) and postmenarchal groups
**Table S2** Characteristics of patients with ovarian cysts and ultrasound approach for visualization, in neonatal (aged < 1 year), premenarchal (aged ≥ 1 year) and postmenarchal groups
**Table S3** Application of simple rules, benign descriptors and expert opinion alone, in neonatal (aged < 1 year), premenarchal (aged ≥ 1 year) and postmenarchal groups
**Table S4** Incidence of uterine pathology per indication for ultrasound: endometrial pathology, congenital uterine anomaly and mean endometrial thickness
**Table S5** Incidence of ovarian pathology per indication for ultrasound: presence of ovarian cyst, diagnosis of ‘polycystic’ ovaries

## Data Availability

Further data that supports the findings of this study are available in the supplementary material of this article. Raw research data are not shared.
