# Supplementary material for: Clinical utility of ultrasonography in pediatric and adolescent gynecology: retrospective review of 1313 ultrasound examinations
Source: Ultrasound Obstet Gynecol. 2025 Feb 2;65(2):226–34. doi: 10.1002/uog.29155 (PMC11788462; doi:10.1002/uog.29155)
Supplement: Supplementary file 1 — Table S1 Findings of ultrasound examination of uterus and ovaries in neonatal (< 1 year), premenarchal (≥ 1 year) and postmenarchal groups Table S2 Characteristics of patients with ovarian cysts and ultrasound approach for visualization, in neonatal (aged < 1 year), premenarchal (aged ≥ 1 year) and postmenarchal groups Table S3 Application of simple rules, benign descriptors and expert opinion alone, in neonatal (aged < 1 year), premenarchal (aged ≥ 1 year) and postmenarchal groups Table S4 Incidence of uterine pathology per indication for ultrasound: endometrial pathology, congenital uterine anomaly and mean endometrial thickness Table S5 Incidence of ovarian pathology per indication for ultrasound: presence of ovarian cyst, diagnosis of ‘polycystic’ ovaries [file UOG-65-226-s001.docx]

Supplementary Information

Table S1 Findings of ultrasound examination of uterus and ovaries of patients, according to neonatal (< 1 year), premenarchal (≥ 1 year) and postmenarchal group

|  | Neonatal | | Pre-menarchal | | Post-menarchal | |
| --- | --- | --- | --- | --- | --- | --- |
|  | **Mean** | **Range** | **Mean** | **Range** | **Mean** | **Range** |
| Uterine dimensions (cm)  Longitudinal  AP  Transverse | 2.4  0.8  1.1 | *1.4 – 4*  *0.3 – 2.5*  *0.8 – 1.6* | *3.7*  *1.5*  *2.3* | *0.8 – 9.6*  *0.4 – 5.0*  *0.7 – 5.3* | *6.5*  *3.1*  *4.3* | *1.6 – 11.1*  *0.5 – 6.7*  *0.9 – 10.1* |
| Uterine volume (ml) | 0.9 | *0.2 – 2.3* | 10.5 | *(4.0 – 70.0)* | 46.8 | *7.0 – 458.0* |
| Endometrial thickness (mm) | 0.5 | *0.0 – 2.0* | 2.3 | *0.0 – 14.6* | 6.3 | *0.0 – 70.0* |
|  | Neonatal | | Pre-menarchal | | Post-menarchal | |
|  | **Mean** | **Range** | **Mean** | **Range** | **Mean** | **Range** |
| Ovarian volume (ml)  Right  Left | 2.5  3.0 | *(0.6 – 5.9)*  *(0.1 – 5.0)* | 4.2  4.8 | *(0.3 – 15.5)*  *(0.1 – 25.4)* | 10.1  9.2 | *(0.5 – 132.0)*  *(0.6 – 122.0)* |

Table S2 Characteristics of patients with ovarian cysts and ultrasound approach for visualization, according to neonatal (aged < 1 year), premenarchal (aged ≥ 1 year) and postmenarchal groups

|  | Neonatal  *(N = 4)* | | Premenarchal  *(N =3)* | | Postmenarchal  *(N = 95)* | |
| --- | --- | --- | --- | --- | --- | --- |
|  | *Median* | *Range* | Median | *Range* | *Median* | *Range* |
| Age at time of first US (years) | 0.0 | *0.0 – 0.0* | 3.8 | *1.0 – 10.2* | 15.7 | *11.1 – 17.9* |
| Maximum cyst diameter (mm) | 37.8 | *10.7 – 78.0* | 19.0 | *11.2 – 102.0* | 43.5 | *13.1 – 108.0* |
| Number of cyst locules (if multilocular) | 2 | *-* | 0 | *0* | 2 | *2 - 6* |
|  | n | *%* | n | *%* | n | *%* |
| Tumor markers performed  Yes  No | 0  4 | *0.0*  *100.0* | 2  1 | *66.7*  *33.3* | 5  90 | *5.3*  *94.7* |
| Locules  Unilocular  Multilocular  Inadequate image | 3  1  0 | *75.0*  *25.0*  *0.0* | 2  0  1 | *66.7*  *0.0*  *33.3* | 82  7  6 | *86.3*  *7.4*  *6.3* |
| Solid components  Yes  No  Uncertain  Inadequate image | 3  0  1  0 | *75.0*  *0.0*  *25.0*  *0.0* | 0  1  1  1 | *0.0*  *33.3*  *33.3*  *33.3* | 0  89  0  6 | *0.0*  *93.7*  *0.0*  *6.3* |
| Cyst content  Anechoic  Hemorrhagic  Mixed  Low level  Groundglass  Uncertain  Inadequate image | 2  1  0  0  0  1  0 | *50.0*  *25.0*  *0.0*  *0.0*  *0.0*  *25.0*  *0.0* | 1  0  1  0  0  0  1 | *33.3*  *0.0*  *33.3*  *0.0*  *0.0*  *33.3*  *33.3* | 43  35  7  0  2  2  6 | *45.3*  *36.8*  *7.4*  *0.0*  *2.1*  *2.1*  *6.3* |
| Cyst walls  Regular  Irregular  Uncertain  Inadequate image | 3  0  1  0 | *75.0*  *0.0*  *25.0*  *0.0* | 1  0  1  1 | *33.3*  *0.0*  *33.3*  *33.3* | 88  1  0  6 | *92.6*  *1.0*  *0.0*  *6.3* |
| IOTA color score  1  Uncertain  Inadequate image | 3  1  0 | *75.0*  *25.0*  *0.0* | 1  1  1 | *33.3*  *33.3*  *33.3* | 87  0  8 | *91.6*  *0.0*  *8.4* |
| Acoustic Shadows  Yes  No  Uncertain  Inadequate image | 3  0  1  0 | *75.0*  *0.0*  *25.0*  *0.0* | 1  0  1  1 | *33.3*  *0*  *33.3*  *33.3* | 7  81  0  7 | *7.4*  *85.3*  *0.0*  *7.4* |
| Free fluid  Yes  No | 0  4 | *0.0*  *100.0* | 0  3 | *0.0*  *100.0* | 7  88 | *7.4*  *92.6* |
| Expert impression of cyst  Benign cystadenoma  Dermoid  Endometrioma  Hemorrhagic  Hydrosalpinx  Paraovarian cyst  Not classified  Inadequate image | 2  0  0  1  0  0  1  0 | *50.0*  *0.0*  *0.0*  *25.0*  *0.0*  *0.0*  *25.0*  *0* | 0  0  0  0  0  0  2  1 | *0.0*  *0.0*  *0.0*  *0.0*  *0.0*  *0.0*  *66.7*  *33.3* | 34  6  2  35  5  4  3  6 | *35.8*  *6.3*  *2.1*  *36.8*  *5.3*  *4.2*  *3.2*  *6.3* |
| Modified Benign Simple Descriptors  Descriptor 1 (endometrioma)  Descriptor 2 (benign cystic teratoma)  Descriptor 3 (simple cyst or cystadenoma)  Descriptor 4 (all unilocular cysts <10cm)  Not classified  Inadequate image | 0  0  2  1  1  0 | *0.0*  *0.0*  *50.0*  *25.0*  *25.0*  *0.0* | 0  0  0  0  2  1 | *0.0*  *0.0*  *0.0*  *0.0*  *66.6*  *33.3* | 2  6  39  35  7  6 | *2.1*  *6.3*  *41.1*  *36.8*  *7.4*  *6.3* |
| Simple Rules  Benign (B only, No M)  Malignant (M only, No B)  Not classifiable  Inadequate image | 3  0  1  0 | *75.0*  *0.0*  *25.0*  *0.0* | 0  0  2  1 | *0*  *0*  *66.7*  *33.3* | 86  0  3  6 | *90.5*  *0.0*  *3.2*  *6.3* |
| Follow up  Discharged  Repeat scan  Speciality follow up (pediatic surgery)  Speciality follow up (gynecology)  Speciality follow up (other)  Not documented | 0  2  2  0  0  0 | *0.0*  *50.0*  *50.0*  *0.0*  *0.0*  *0.0* | 1  0  1  0  1  0 | *33.3*  *0.0*  *33.3*  *0.0*  *33.3*  *0.0* | 11  62  1  19  1  1 | *11.6*  *65.3*  *1.1*  *20.0*  *1.1*  *1.1* |
| Additional imaging performed  Yes  No  Recommended but not performed | 1  3  0 | *25.0*  *75.0*  *0.0* | 3  0  0 | *100.0*  *0.0*  *0.0* | 8  86  1 | *8.4*  *90.5*  *1.1* |
| Surgical management after 1^st^ scan  Yes  No | 0  4 | *0.0*  *100.0* | 1  2 | *33.3*  *66.7* | 5  90 | *5.3*  *94.7* |
| Repeat scan findings (n/N)  Resolution  Increased in size  New pathology  No change  Other  Lost to follow up  Referred to other center for scan | (4/4)  1  0  0  0  1  2  0 | *(100)*  *25.0*  *0.0*  *0.0*  *0.0*  *25.0*  *50.0*  *0.0* | (2/3)  0  0  0  0  1  0  1 | *(66.6)*  *0.0*  *0.0*  *0.0*  *0.0*  *50.0*  *0.0*  *50.0* | (62/95)  40  3  1  11  2  5  0 | *(65.2)*  *64.5*  *4.8*  *1.6*  *17.7*  *3.2*  *8.1*  *0.0* |

IOTA, International Ovarian Tumour Analysis; US, ultrasound. Percentages calculated for which data were available.

Table S3: Application of simple rules, benign descriptors and expert opinion alone, according to neonatal (aged < 1 year), premenarchal (aged ≥ 1 year) and postmenarchal groups

|  | Neonatal (n = 4) | | | Pre-menarchal (n = 3) | | | Post-menarchal (n = 95) | | |
| --- | --- | --- | --- | --- | --- | --- | --- | --- | --- |
| Simple Rules  Predicted Benign: Confirmed Benign  Not Classifiable: Confirmed Benign  Predicted Malignant: Confirmed Malignant  Not Classifiable: Confirmed Malignant  No outcome  Modified Benign Descriptors  Described Benign: Confirmed benign  Described Benign: Confirmed malignant  Not Applicable: Confirmed Benign  No outcome  Expert Opinion  Predicted Benign: Confirmed Benign  Not Classified: Confirmed Benign  Predicted Malignant: Confirmed Malignant  Not Classifiable: Confirmed Malignant  No outcome  Standard Care  Predicted Benign: Confirmed Benign  Incorrectly/Not Classified: Confirmed Benign  Predicted Malignant: Confirmed Malignant  Incorrectly/Not Classified: Confirmed Malignant  No outcome | N  1  1  0  0  2  1  0  1  2  1  1  0  0  2  0  2  0  0  2 | *% (all)*  *25.0*  *25.0*  *0.0*  *0.0*  *50.0*  *25.0*  *0.0*  *25.0*  *50.0*  *25.0*  *25.0*  *0.0*  *0.0*  *50.0*  *0.0*  *50.0*  *0.0*  *0.0*  *50.0* | % (Excluding ‘no outcome’)  50.0  50.0  0.0  0.0  -  50.0  0.0  50.0  -  50.0  50.0  0.0  0.0  -  0.0  100%  0.0  0.0  - | N  0  2  0  0  1  0  0  2  1  0  2  0  0  1  0  2  0  0  1 | *% (all)*  *0.0*  *66.6*  *0.0*  *0.0*  *33.3*  *0.0*  *0.0*  *66.6*  *33.3*  *0.0*  *66.6*  *0.0*  *0.0*  *33.3*  *0.0*  *66.6*  *0.0*  *0.0*  *33.3* | % (Excluding ‘no outcome’)  *0.0*  *100.0*  *0.0*  *0.0*  *-*  *0.0*  *0.0*  *100.0*  *-*  *0.0*  *100.0*  *0.0*  *0.0*  *-*  *0.0*  *100.0*  *0.0*  *0.0*  *-* | N  76  3  0  0  16  69  0  10  16  78  1  0  0  16  61  18  0  0  16 | *% (all)*  *80.0*  *3.2*  *0.0*  *0.0*  *16.8*  *72.6*  *0.0*  *10.5*  *16.8*  *82.1*  *1.1*  *0.0*  *0.0*  *16.8*  *64.2*  *18.9*  *0.0*  *0.0*  *16.8* | % (Excluding ‘no outcome’)  *96.2*  *3.8*  *0.0*  *0.0*  *-*  *87.3*  *0.0*  *12.7*  *-*  *98.7*  *1.3*  *0.0*  *0.0*  *-*  *77.2*  *22.8*  *0.0*  *0.0*  *-* |

Table S4: Incidence of uterine pathology per indication for ultrasound: endometrial pathology, congenital uterine anomaly and mean endometrial thickness

| INDICATION FOR USS | ENDOMETRIAL PATHOLOGY | | | | | | | | | | | | |
| --- | --- | --- | --- | --- | --- | --- | --- | --- | --- | --- | --- | --- | --- |
|  | **NEONATAL** | | | | **PRE-MENARCHAL** | | | | **POST-MENARCHAL** | | | | |
|  | **Normal** | | **Pathology Seen** | | **Normal** | | **Pathology Seen** | | **Normal** | | **Pathology Seen** | | |
|  | n | *%* | n | *%* | n | *%* | n | *%* | n | *%* | n | *%* |  |
| Abnormal PV bleeding (all)  *Menorrhagia*  *Oligomenorrhoea*  *Other (IMB/PCB/pre-pubertal)*  Abdominal pain  Primary amenorrhoea  Secondary amenorrhoea  Known medical condition  Change in bowel habit  Precocious puberty  Uncertainty of menarchal status  Hirsutism  Not documented  Other | 1  -  -  *1*  -  -  -  10  -  -  -  -  -  8 | *5.3*  *-*  *-*  *5.3*  *-*  *-*  *-*  *52.6*  *-*  *-*  *-*  *-*  *-*  *42.1* | 0  -  -  0  -  -  -  0  -  -  -  -  -  0 | *0.0*  *-*  *-*  *0.0*  *-*  *-*  *-*  *0.0*  *-*  *-*  *-*  *-*  *-*  *0.0* | 10  -  -  *10*  8  25  -  4  -  37  1  1  4  22 | *8.8*  *-*  *-*  *8.8*  *7.0*  *21.9*  *-*  *3.5*  *-*  *32.5*  *0.9*  *0.9*  *3.5*  *19.3* | 0  -  *-*  *0*  1  1  -  0  -  0  0  0  0  0 | *0.0*  *-*  *-*  *0.0*  *0.9*  *0.9*  *-*  *0.0*  *-*  *0.0*  *0.0*  *0.0*  *0.0*  *0.0* | 485  *196*  *257*  *32*  181  -  62  25  1  -  1  20  68  79 | *51.1*  *20.7*  *27.1*  *3.4*  *19.1*  *-*  *6.5*  *2.6*  *0.1*  *-*  *0.1*  *2.0*  *7.2*  *8.3* | 15  8  4  3  5  -  0  3  0  -  0  0  1  3 | *1.6*  *0.8*  *0.4*  *0.3*  *0.5*  *-*  *0.0*  *0.3*  *0.0*  *-*  *0.0*  *0.0*  *0.1*  *0.3* |  |
| INDICATION FOR USS | **UTERINE ANOMALY** | | | | | | | | | | | | |
|  | **NEONATAL** | | | | **PRE-MENARCHAL** | | | | **POST-MENARCHAL** | | | | |
|  | **Normal** | | **Anomaly Seen** | | **Normal** | | **Anomaly Seen** | | **Normal** | | **Anomaly Seen** | | |
|  | n | *%* | n | *%* | n | *%* | n | *%* | n | *%* | n | *%* |  |
| Abnormal PV bleeding (all)  *Menorrhagia*  *Oligomenorrhoea*  *Other (IMB/PCB/pre-pubertal)*  Abdominal pain  Primary amenorrhoea  Secondary amenorrhoea  Known medical condition  Change in bowel habit  Precocious puberty  Uncertainty of menarchal status  Hirsutism  Not documented  Other | 1  -  -  *1*  -  -  -  11  -  -  -  -  -  8 | *5.0*  *-*  *-*  *5.0*  *-*  *-*  *-*  *55.0*  *-*  *-*  *-*  *-*  *-*  *45.0* | 0  -  -  0  -  -  -  0  -  -  -  -  -  0 | *0.0*  *-*  *-*  *0.0*  *-*  *-*  *-*  *0.0*  *-*  *-*  *-*  *-*  *-*  *0.0* | 11  -  1  10  10  28  -  4  -  38  2  1  4  23 | *8.9*  *-*  *0.8*  *8.1*  *8.1*  *22.6*  *-*  *3.2*  *-*  *30.6*  *1.6*  *0.8*  *3.2*  *18.5* | 0  -  0  0  0  3  -  0  -  0  0  0  0  0 | *0.0*  *-*  *0.0*  *0.0*  *0.0*  *2.4*  *-*  *0.0*  *-*  *0.0*  *0.0*  *0.0*  *0.0*  *0.0* | 500  *203*  *262*  *35*  184  -  63  28  1  -  2  20  68  85 | *52.0*  *21.1*  *27.3*  *3.6*  *19.1*  *-*  *6.6*  *2.9*  *0.1*  *-*  *0.2*  *2.1*  *70.8*  *8.8* | 4  3  1  0  3  -  0  1  0  -  1  0  1  0 | *0.4*  *0.3*  *0.1*  *0.0*  *0.3*  *-*  *0.0*  *0.1*  *0.0*  *-*  *0.1*  *0.0*  *0.1*  *0.0* |  |
| INDICATION FOR USS | **ENDOMETRIAL THICKNESS (MM)** | | | | | | | | | | | | |
|  | **NEONATAL** | | | | **PRE-MENARCHAL** | | | | **POST-MENARCHAL** | | | | |
|  | **n** | | **Mean ET (range)** | | **n** | | **Mean ET (range)** | | **n** | | **Mean ET (range)** | | |
| Abnormal PV bleeding  *Menorrhagia*  *Oligomenorrhoea*  *Other (IMB/PCB/pre-pubertal)*  Abdominal pain  Primary amenorrhoea  Secondary amenorrhoea  Known medical condition  Change in bowel habit  Precocious puberty  Uncertainty of menarchal status  Hirsutism  Other | *-*  *-*  *1*  -  -  -  7  -  -  -  -  4 | | *-*  *-*  *0.0*  *-*  *-*  *-*  *0.7 (0.0 – 2.0)*  *-*  *-*  *-*  *-*  *0.4 (0.0 – 1.5)* | | *-*  *-*  *9*  *6*  *23*  *-*  *3*  *-*  *23*  *2*  *-*  *16* | | *-*  *-*  *0.8 (0.0 – 2.0)*  *1.2 (0 – 4.2)*  *4.8 (0 – 14.6)*  *-*  *0.0 (0.0)*  *-*  *1.5 (0.0 – 7.2)*  *5.6 (3.2 – 8.0)*  *-*  *1.5 (0.0 – 10.0)* | | *193*  *249*  *35*  *165*  *-*  *58*  *23*  *1*  *-*  *2*  *-*  *75* | | *7.0 (1.0 – 26.0)*  *6.5 (0.0 – 18.0)*  *6.7 (2.0 – 16.0)*  *6.4 (0.0 – 23.0)*  *-*  *5.6 (0.0 – 12.8)*  *10.6 (1.0 – 70.0)*  *10*  *-*  *3.4 (0.0 – 6.7)*  *-*  *6.5 (0.9 – 16.0)* | | |

Table S5: Incidence of ovarian pathology per indication for ultrasound: presence of ovarian cyst, diagnosis of ‘polycystic’ ovaries

| INDICATION FOR USS | OVARIAN PATHOLOGY (CYST) | | | | | | | | | | | | |
| --- | --- | --- | --- | --- | --- | --- | --- | --- | --- | --- | --- | --- | --- |
|  | **NEONATAL** | | | | **PRE-MENARCHAL** | | | | **POST-MENARCHAL** | | | | |
|  | **Normal** | | **Cyst Seen** | | **Normal** | | **Cyst Seen** | | **Normal** | | **Cyst Seen** | | |
|  | **n** | ***%*** | **n** | ***%*** | **n** | ***%*** | **n** | ***%*** | **n** | ***%*** | **n** | ***%*** |  |
| Abnormal PV bleeding (all)  *Menorrhagia*  *Oligomenorrhoea*  *Other (IMB/PCB/pre-pubertal)*  *Uncertainty of m*  Abdominal pain  Primary amenorrhoea  Secondary amenorrhoea  Known medical condition  Change in bowel habit  Precocious puberty  Uncertainty of menarchal status  Hirsutism  Not documented  Other | 1  -  -  1  -  -  -  8  -  -  -  -  -  7 | 5.0  -  -  5.0  -  -  -  40.0  -  -  -  -  -  35.0 | 0  -  -  0  -  -  -  3  -  -  -  -  -  1 | 0.0  -  -  0.0  -  -  -  15.0  -  -  -  -  -  5.0 | 9  -  -  9  10  31  -  3  -  38  2  1  4  23 | *7.3*  *-*  *-*  *7.3*  *8.1*  *25.0*  *-*  *2.4*  *-*  *3.1*  *1.6*  *0.8*  *3.2*  *18.5* | 1  -  -  1  0  0  -  1  -  0  0  0  0  1 | *0.01*  *-*  *-*  *0.8*  *0.0*  *0.0*  *-*  *0.8*  *-*  *0.0*  *0.0*  *0.0*  *0.0*  *0.8* | 464  180  249  33  164  -  62  20  1  -  3  19  61  74 | *48.2*  *18.7*  *25.9*  *3.4*  *17.1*  *-*  *6.5*  *2.1*  *0.1*  *-*  *0.3*  *19.8*  *6.3*  *7.7* | 42  26  14  2  23  -  1  9  0  -  0  1  8  11 | *4.4*  *2.7*  *1.5*  *0.2*  *2.4*  *-*  *0.8*  *9.4*  *0.0*  *-*  *0.0*  *0.1*  *0.8*  *1.1* |  |
| INDICATION FOR USS | LABELLED POLYCYSTIC | | | | | | | | | | | | |
|  | **NEONATAL** | | | | **PRE-MENARCHAL** | | | | **POST-MENARCHAL** | | | | |
|  | Normal | | PCO | | Normal | | PCO | | Normal | | PCO | | |
|  | n | *%* | n | *%* | n | *%* | n | *%* | n | *%* | n | *%* |  |
| Abnormal PV bleeding (all)  *Menorrhagia*  *Oligomenorrhoea*  *Other (IMB/PCB/pre-pubertal)*  Abdominal pain  Primary amenorrhoea  Secondary amenorrhoea  Known medical condition  Change in bowel habit  Precocious puberty  Uncertainty of menarchal status  Hirsutism  Not documented  Other | 1  -  -  1  -  -  -  11  -  -  -  -  -  8 | 5.0  -  -  5.0  -  -  -  55.0  -  -  -  -  -  40.0 | 0  -  -  0  -  -  -  0  -  -  -  -  -  0 | 0.0  -  -  0.0  -  -  -  0.0  -  -  -  -  -  0.0 | 10  -  -  10  10  26  -  4  -  38  1  1  5  23 | *8.1*  *-*  *-*  *8.1*  *8.1*  *21.0*  *-*  *3.2*  *-*  *30.6*  *0.8*  *0.8*  *4.0*  *18.5* | 0.0  -  -  0  0  5  -  0  -  0  1  0  0  0 | 0.0  -  -  0.0  0.0  4.0  -  0.0  -  0.0  0.8  0.0  0.0  0.0 | 409  186  195  28  177  -  48  27  1  -  3  13  11  75 | *42.6*  *19.4*  *20.3*  *2.9*  *18.4*  *-*  *5.0*  *2.8*  *0.1*  *-*  *3.1*  *1.4*  *1.1*  *7.8* | 95  20  68  7  10  -  15  2  0  -  0  7  58  10 | *9.9*  *2.1*  *7.1*  *0.7*  *1.0*  *-*  *1.6*  *0.2*  *0.0*  *-*  *0.0*  *0.7*  *6.0*  *1.0* |  |
